# Professional Identity as a Mechanism Linking Moral Sensitivity to Moral Courage Among Clinical Nurses: A Cross‐Sectional Study

**DOI:** 10.1155/jonm/7839527

**Published:** 2026-09-29

**Authors:** Yuanyuan Zhang, Yujing Wang

**Affiliations:** ^1^ School and Hospital of Stomatology Liaoning Provincial Key Laboratory of Oral Diseases China Medical University Shenyang China cmu.edu.tw

**Keywords:** moral courage, moral sensitivity, night shift work, nursing management, patient advocacy, professional identity

## Abstract

**Aim:**

This study examined whether professional identity mediates the association between moral sensitivity and moral courage among clinical nurses.

**Background:**

Moral courage enables nurses to advocate for patients in ethically challenging situations, yet nurse managers lack evidence‐based guidance on how to cultivate this competency. While moral sensitivity is a known antecedent of moral courage, the mechanisms translating recognition into action remain unclear.

**Methods:**

A cross‐sectional survey of 1049 clinical nurses was conducted at two tertiary hospitals in Dalian, China (March 22–April 9, 2026). Participants completed validated measures of moral sensitivity, professional identity, and moral courage. Mediation analysis used PROCESS Macro Model 4 with 5000 bootstrap resamples.

**Results:**

Moral sensitivity was positively correlated with professional identity (*r* = 0.333, *p* < 0.001) and moral courage (*r* = 0.517, *p* < 0.001). Professional identity partially mediated this relationship, accounting for 26.38% (95% CI: 0.298–0.473) of the total effect. Head nurses reported significantly higher moral courage scores than staff nurses (*p* < 0.001). Operating room nurses had the lowest scores (mean = 68.21, SD = 8.53), and nurses working ≥ 5 night shifts per month scored significantly lower than those working 0 or 1–4 shifts (*p* < 0.001).

**Conclusion:**

Concurrent associations suggest professional identity accounts for approximately one‐quarter of the correlational linkage between moral sensitivity and self‐reported moral courage in this sample. This concurrent pattern implies that nurses’ capacity to identify clinical ethical issues does not inherently align with self‐reported willingness to take ethical action. Based solely on cross‐sectional correlational evidence, nursing leaders may tentatively regard professional identity development as a potential direction for workplace ethics support activities rather than a definitive intervention target.

**Implications for Nursing Management:**

Preliminary practice directions derived from these correlational patterns are as follows. Nursing administrators may integrate reflective activities focused on nursing professional values into standard ethics training curricula. Customized ethical support resources can be offered to two subgroups with lower average moral courage scores: operating room nurses and staff completing five or more monthly night rotations. Institutional informal acknowledgment mechanisms for nurses’ ethical advocacy conduct may also be explored, though all above suggestions require longitudinal validation before formal implementation.

## 1. Background

Nurse managers worldwide face a persistent challenge: how to support a workforce capable of ethical action in high‐stakes clinical environments. For clinical nurses, moral courage represents the ability to sustain ethical commitments amid difficult clinical circumstances, despite potential negative personal‐ or career‐related consequences. This capability underpins patient‐oriented advocacy and may buffer nurses from moral distress and progressive erosion of ethical practice standards. [1–4]. Yet evidence suggests considerable variability in moral courage levels across nursing populations [5, 6], and nurse managers lack systematic guidance on how to foster this competency within their teams. Understanding the modifiable antecedents of moral courage is therefore an urgent priority for nursing management research.

From ethical psychology perspectives, identifying clinical ethical dilemmas (moral sensitivity) functions as a prerequisite psychological correlate of self‐reported ethical action; nurses cannot display courageous advocacy if they fail to detect workplace ethical conflicts in routine care [7, 8]. Empirical support for this premise has accumulated in recent years. In a sample of 465 Chinese clinical nurses, Chen and colleagues documented a positive association between moral sensitivity scores and self‐reported ethical behavior [9]. Similarly, Luo et al. reported significant correlations between moral sensitivity and moral courage among nurse interns, with higher sensitivity scores predicting greater willingness to confront ethical challenges [8]. These findings suggest that nurses who more readily identify ethical nuances in patient care situations are subsequently better positioned to respond with courageous action [10].

However, the pathway from moral sensitivity to moral courage is unlikely to be direct or unmediated [11]. Psychological theories of ethical action emphasize that recognizing an ethical issue (sensitivity) is insufficient; individuals must also possess a sense of personal agency and professional commitment before they will risk adverse consequences to act [12, 13]. For nurse managers, this theoretical insight raises a practical question: Given that ethical awareness rarely shows a standalone concurrent correlation with nurses’ willingness to take ethical risks, this study explores concurrent psychological correlates that co‐occur alongside both constructs for managerial reference. Identifying such mechanisms is essential for designing evidence‐based management strategies, yet empirical research in this area remains sparse. This insight directs attention toward professional identity, defined as the internalized sense of belonging to and alignment with the values of the nursing profession [14]. To develop empirically testable correlational hypotheses, the present study draws upon Rest’s four‐component model of ethical functioning as its primary theoretical framework [15, 16]. This model describes four interconnected phases shaping nurses’ ethical responses: (1) moral sensitivity—recognizing that an ethical issue exists; (2) moral judgment—determining the most ethically justifiable course of action; (3) moral motivation—prioritizing ethical values above competing personal or professional demands; and (4) moral implementation—possessing the perseverance to execute the chosen action despite obstacles [17]. Within this framework, professional identity corresponds to moral motivation—the psychological mechanism that compels nurses to prioritize professional ethical values amid workplace pressures [18, 19]. A well‐developed professional identity helps nurses reinterpret ethical challenges not merely as personal predicaments but as concerns tied to their professional role [20], thereby lowering the psychological barriers to ethically courageous conduct [21, 22].

Several studies have supported this reasoning. Among 800 Chinese nurses, moral courage partially mediated the relationship between moral distress and career identity, suggesting that identity and courage are dynamically interrelated [23]. In a recent study conducted among Turkish nurses, Köktürk Dalcali and colleagues found a positive and moderate correlation between professional values and moral courage (*r* = 0.541, *p* < 0.001), with professional values emerging as a strong determinant of nurses’ moral courage [24]. Although professional values and professional identity are conceptually distinct, they are closely intertwined: professional identity inherently involves the internalization of professional values [14]. Accordingly, these findings indirectly support the premise that professional identity plays a pivotal role in enabling nurses to act on their ethical convictions.

Notably, the theoretical possibility that professional identity mediates the relationship between moral sensitivity and moral courage remains largely untested. Although moral sensitivity and professional identity have each been examined separately in relation to moral courage [25–27], the underlying mechanisms through which these three constructs interact remain unclear. Exploring this underexamined concurrent indirect pathway carries tentative theoretical and managerial value. If professional identity co‐varies alongside the linkage between moral sensitivity and moral courage, workplace activities aligned with nurses’ professional value identification may concurrently correlate with elevated ethical awareness outcomes in cross‐sectional observation.

The present study was designed to test this mediation model by testing a mediation model with direct implications for nursing management. Specifically, we tested four hypotheses:

 H1: Moral sensitivity is positively associated with moral courage.
 H2: Moral sensitivity is positively associated with professional identity.
 H3: Professional identity is positively associated with moral courage.
 H4: Professional identity mediates the relationship between moral sensitivity and moral courage.

The four correlational hypotheses proposed in this analysis aim to quantify direct and indirect concurrent relationships among moral sensitivity, professional identity, and moral courage among Chinese front‐line nurses. Findings are intended to generate tentative directional insights for nursing administrative practice.

## 2. Methods

### 2.1. Study Participants and Data Collection

A cross‐sectional survey was conducted between March 22 and April 9, 2026 using convenience sampling. Nurses were recruited from two tertiary hospitals in Dalian, Liaoning Province, China. Participants were included if they: (a) held a valid nursing practice license; (b) were aged 18 to 60 years; (c) had at least 6 months of clinical front‐line experience; and (d) provided voluntary written informed consent. Nurses were excluded if they: (a) were undergoing advanced training, internships, or standardized residency programs without independent practice status; (b) were on extended leave due to illness or maternity leave during the data collection period; (c) were unwilling or unable to complete the questionnaire; (d) were engaged in administrative, teaching, or other nonclinical nursing roles during the data collection period. The questionnaire was administered online via the Wenjuanxing platform. Before initiating the survey, all potential participants were provided with information about the study purpose and procedures. Written informed consent was obtained from each participant through the online platform before proceeding to the questionnaire. Participants were informed that they could withdraw from the study at any time without any consequences.

A total of 1216 questionnaires were distributed. Four respondents declined to participate, and seven were excluded because their reported age values were outside the prespecified range of 18 to 60 years. After removing 156 invalid questionnaires that contained duplicate response patterns, a total of 1049 valid questionnaires were retained for final analysis, yielding an effective response rate of 86.27%.

Sample size calculation followed established mediation analytical guidance, which recommends a minimum of 5–10 respondents for each estimated research parameter [28]. With a total of 46 scale items included in the survey, the preliminary target sample fell between 230 and 460 participants. We expanded the target sample range to 253–506 to accommodate an expected 10% proportion of incomplete or repetitive invalid questionnaires.

### 2.2. Ethical Considerations

Ethical approval was obtained from the Ethics Committee of the Hospital of Stomatology, China Medical University (Approval No.: K2026007). All procedures performed were in accordance with the ethical standards of the institutional and national research committee and with the 1964 Helsinki Declaration and its later amendments. Written informed consent was obtained from each participant through the online platform before proceeding to the questionnaire. Participation was voluntary, and confidentiality of the data was strictly maintained throughout the study.

### 2.3. Measures

#### 2.3.1. Demographic Questionnaire

A self‐designed demographic questionnaire was used to collect participants’ basic information, including age, gender, educational background, professional title, position, department, employment type, and average number of night shifts per month. The specific categorical options for each variable remain detailed in Table 1.

**TABLE 1:** Sample characteristics and differences in moral courage scores (*N* = 1049).

Variables	*N* (%)	Moral courage				
		(M ± SD)	t/F	*p*	Post hoc test	*p*
Age (year)			0.766	0.513		
21–30	295 (28.12)	77.27 ± 16.30				
31–40	503 (47.95)	77.94 ± 15.30				
41–50	204 (19.45)	78.47 ± 13.77				
≥ 51	47 (4.48)	80.68 ± 16.06				
Gender			−0.989	0.323		
Male	35 (3.34)	75.46 ± 14.77				
Female	1014 (96.66)	78.06 ± 15.35				
Marital status			1.460	0.233		
Single	304 (28.98)	77.19 ± 15.51				
Married	729 (69.49)	78.19 ± 15.22				
Others	16 (1.53)	83.38 ± 16.75				
Education			1.871	0.154		
Junior college	61 (5.82)	75.90 ± 16.16				
Undergraduate	952 (90.75)	77.95 ± 15.31				
Master	36 (3.43)	82.11 ± 14.20				
Professional title			2.427	0.064		
Nurse	72 (6.86)	77.65 ± 15.83				
Senior nurse	302 (28.79)	76.56 ± 15.86				
Supervisor nurse	538 (51.29)	78.10 ± 15.31				
Associate director	137 (13.06)	80.79 ± 13.68				
Position			−4.719	0.000**^∗∗∗^**		
Staff nurse	944 (89.99)	77.24 ± 15.31				
Head nurse	105 (10.01)	84.61 ± 13.96				
Department			2.546	0.010**^∗^**	④ > ①, ②, ③, ⑤, ⑥, ⑦; ⑦ < ①, ②, ④, ⑧, ⑨	
①Internal medicine	419 (39.94)	78.53 ± 15.62			① < ④,	0.032
					① > ⑦	0.004
②Surgery	212 (20.21)	77.52 ± 15.13			② < ④	0.013
					② > ⑦	0.011
③ Gynecology	35 (3.34)	76.49 ± 16.90			③ < ④	0.047
④Paediatrics	70 (6.67)	82.76 ± 15.01			④ > ⑥	0.002
⑤Emergency	47 (4.48)	75.47 ± 14.41			⑤ < ④	0.011
⑥ICU	69 (6.58)	74.81 ± 16.15			⑥ < ④	0.002
⑦Operating room	19 (1.81)	68.21 ± 8.53			⑦ < ④	0.000
⑧Outpatient	85 (8.10)	78.93 ± 14.62			⑧ > ⑦	0.006
⑨Others	93 (8.87)	78.23 ± 14.60			⑨ > ⑦	0.009
Employment type			1.648	0.100		
Permanent	265 (25.26)	79.29 ± 14.52				
Contract	784 (74.74)	77.50 ± 15.57				
Average night shifts/month			12.770	**<** **0.001**		
0	374 (35.65)	79.07 ± 14.67			① < ②	0.055
1–4	202 (19.26)	81.61 ± 14.44			② > ③	0.000
≥ 5	473 (45.09)	75.56 ± 15.34			③ < ①	0.001

*Note:* The ‘Others’ category under department includes digestive endoscopy center, bronchoscopy unit, interventional catheterization unit, hyperbaric oxygen unit, blood purification center, and so on. Bold values indicate statistically significant coefficients (*p* < 0.05).

Abbreviation: SD, standard deviation.

^∗^ *p* < 0.05.

^∗∗∗^ *p* < 0.001 (two‐tailed).

#### 2.3.2. Moral Sensitivity Questionnaire

Nurses’ capacity to detect clinical ethical conflicts was measured via the revised Moral Sensitivity Questionnaire (MSQ‐R). The original tool was created by Lützén et al. in 2006 [29], and Huang et al. completed its cross‐cultural localization and reliability testing for Chinese nurse populations in 2016 [30]. The localized scale contains nine measurement items split into two subdimensions: moral strength and responsibility (items 1, 2, 3, 5, 9) and moral burden (items 4, 6, 7, 8). Responses were captured on a six‐point Likert‐type scale, with response choices: ‘strongly disagree’ (score 1), ‘disagree’ (score 2), ‘somewhat disagree’ (score 3), ‘somewhat agree’ (score 4), ‘agree’ (score 5), and ‘strongly agree’ (score 6). Total scores are computed as the sum across all nine items, yielding possible scores from 9 to 54; higher total scores correspond to greater moral sensitivity among participating nurses. The adapted scale has been shown to be applicable for measuring moral sensitivity in Chinese nurses [31]. In the current work, the Cronbach’s α coefficient for the scale was 0.878.

#### 2.3.3. Moral Courage Scale

Our investigation used the Chinese version of the Nurse Moral Courage Scale (NMCS), which was translated and culturally adapted by Wang et al. [32]. The original scale was developed by Nummine et al. [33] to measure clinical nurses’ self‐assessed moral courage. The scale consists of 21 items across four dimensions: Moral integrity (7 items), commitment to providing good patient care (5 items), compassion and genuine presence with patients (5 items), moral responsibility (4 items). Every item uses a five‐point Likert‐type response scale, anchored by ‘completely inconsistent with me’ (1) and ‘completely consistent with me’ (5). Possible total scores span 21–105; higher scores reflect greater self‐reported moral courage in clinical work. The Chinese version of the NMCS has been widely used [5]. In this study, the Cronbach’s α coefficient of the scale was 0.955.

#### 2.3.4. Nurses’ Professional Identity Scale

In this study, the Nurse Professional Identity Scale developed by Liu et al. [34] was used to assess the level of professional identity among newly recruited nurses. The scale consists of 30 items across five dimensions: professional cognitive evaluation (items 1–9), professional social skills (items 10–15), professional social support (items 16–21), professional frustration coping (items 22–27), and professional self‐reflection (items 28–30). Items were answered using a five‐point Likert‐type scale: ‘strongly disagree’, ‘disagree’, ‘occasionally agree’, ‘agree’, ‘strongly agree’, corresponding to scores of 1 through 5. Total possible scores vary between 30 and 150, where larger scores denote stronger professional identity. This scale has been widely validated in the Chinese context [35]. In this study, the Cronbach’s α coefficient of the scale was 0.976.

### 2.4. Data Analysis

Statistical analyses were performed using SPSS version 26.0 and the PROCESS Macro. Categorical demographic variables were summarized using frequencies and percentages. Independent‐samples *t*‐tests and one‐way analysis of variance (ANOVA) were used to examine differences in moral courage scores across demographic subgroups, as appropriate. When the ANOVA result was statistically significant, post hoc comparisons were conducted using the least significant difference (LSD) method to identify which specific groups differed from each other.

Pearson correlation analysis was conducted to examine bivariate relationships among moral sensitivity, professional identity, and moral courage. Continuous variables were summarized as mean ± standard deviation (SD).

We applied Hayes’ PROCESS Macro Model 4 to test whether professional identity exerted a mediating effect linking moral sensitivity to moral courage. Bootstrapping with 5000 resamples was used to generate estimates for the indirect effect. Statistical significance for the mediating pathway was established at α = 0.05 if the 95% bias‐corrected and accelerated (BCa) bootstrap confidence interval (CI) for the indirect effect excluded zero.

To control for potential confounding effects, position, department, and average night shifts per month were entered as covariates in the mediation model. Three demographic characteristics were set as confounding control variables within the mediation model via dummy variable transformation. Occupational position was dichotomized, with general ward staff nurses coded as the reference group and head nurses assigned a value of 1. Internal medicine was selected as the reference category for departmental dummy variables. For monthly night rotation frequency, participants with zero night shifts formed the reference group, split into two contrast groups: 1–4 monthly shifts and five or more monthly shifts.

We conducted Harman’s unrotated single‐factor exploratory factor analysis to evaluate common method variance stemming from single‐time‐point self‐report data [36]. All items from the three measurement instruments were entered into factor extraction procedures. The largest single extracted factor explained 42.45% of overall total variance. Per widely accepted methodological benchmarks, a 50% cut‐off value defines severe common method bias, while several behavioral science studies apply a stricter 40% threshold [37]. The present 42.45% value sits under the primary 50% standard and only slightly surpasses the stricter cutoff, suggesting common‐method variance is unlikely to substantially distort the observed correlational patterns, although it cannot be fully excluded.

## 3. Results

### 3.1. Demographic Characteristics of Participants

A total of 1049 valid questionnaires were included in the final analysis (effective rate: 86.27%). The demographic characteristics of the participants are presented in Table 1.

Significant differences in moral courage scores were found based on position (*p* < 0.001), department (*p* = 0.010), and average night shifts per month (*p* < 0.001). Post hoc comparisons were performed for department and night shift frequency using the LSD method.

Head nurses reported significantly higher moral courage scores than staff nurses (mean = 84.61, SD = 13.96 vs. mean = 77.24, SD = 15.31; *p* < 0.001). Among departments, operating room nurses had the lowest moral courage scores (mean = 68.21, SD = 8.53), significantly lower than those in internal medicine (*p* = 0.004), paediatrics (*p* < 0.001), outpatient (*p* = 0.006), and other departments (*p* = 0.009). Paediatrics nurses had the highest scores, significantly higher than surgery (*p* = 0.013), emergency (*p* = 0.011), ICU (*p* = 0.002), and operating room (*p* < 0.001).

For night shifts, nurses working 1–4 shifts per month had the highest moral courage scores (mean = 81.61, SD = 14.44), significantly higher than those working 5 or more shifts (mean = 75.56, SD = 15.34; *p* < 0.001). Nurses working 5 or more shifts scored significantly lower than those working no shifts (mean = 79.07, SD = 14.67; *p* = 0.001). The difference between 1–4 shifts and no shifts was not statistically significant (*p* = 0.055).

### 3.2. Descriptive Statistics of Study Variables

Descriptive statistics for moral sensitivity, professional identity, and moral courage are presented in Table 2. The mean total score for moral sensitivity was 39.45 (SD = 5.37), with a mean item score of 4.38 (SD = 0.60) on a 6‐point scale, indicating a relatively high level of moral sensitivity among participants. The mean total score for professional identity was 113.43 (SD = 20.66), with a mean item score of 3.78 (SD = 0.69) on a 5‐point scale. The mean total score for moral courage was 77.98 (SD = 15.34), with a mean item score of 3.73 (SD = 0.78) on a 5‐point scale. Among the four subscales of moral courage, compassionate presence yielded the highest mean item score (*M* = 3.85, SD = 0.82), whereas commitment to quality patient care yielded the lowest (*M* = 3.53, SD = 0.88).

**TABLE 2:** Total scores and mean item scores of moral sensitivity, professional identity, and moral courage (*N* = 1049).

Variables	Min	Max	Total score		Mean item score	
			Mean	SD	Mean	SD
Moral sensitivity	9	54	39.45	5.37	4.38	0.60
Professional identity	38	150	113.43	20.66	3.78	0.69
Moral courage	23	105	77.98	15.34	3.73	0.78
Moral integrity	9	35	26.27	5.18	3.75	0.74
Commitment to quality patient care	5	25	17.53	4.19	3.53	0.88
Compassionate presence	5	25	19.18	3.87	3.85	0.82
Moral responsibility	4	20	15.00	3.19	3.84	0.81

*Note:* SD, standard deviation.

### 3.3. Bivariate Correlations Among Moral Sensitivity, Professional Identity, and Moral Courage

Pearson correlation coefficients were computed to examine the bivariate relationships among moral sensitivity, professional identity, and moral courage (Table 3). Moral sensitivity was positively and significantly correlated with professional identity (*r* = 0.333, *p* < 0.001) and with moral courage (*r* = 0.517, *p* < 0.001). Professional identity was also positively and significantly correlated with moral courage (*r* = 0.540, *p* < 0.001). The correlation between professional identity and moral courage was stronger than that between moral sensitivity and professional identity.

**TABLE 3:** Correlation analysis among moral sensitivity, professional identity, and moral courage (*N* = 1049).

Variables	Moral sensitivity	Professional identity	Moral courage
Moral sensitivity	1.000		
Professional identity	0.333^∗∗∗^	1.000	
Moral courage	0.517^∗∗∗^	0.540^∗∗∗^	1.000

^∗∗∗^ *p* < 0.001 (two‐tailed).

### 3.4. Decomposition and Mediating Effect Test of Professional Identity

Mediation analysis was conducted using PROCESS Macro Model 4 to test whether professional identity mediated the relationship between moral sensitivity and moral courage, with position, department, and average night shifts per month entered as covariates. The results are presented in Tables 4 and 5.

**TABLE 4:** Regression analysis results for the mediating role of professional identity (*N* = 1049).

Variable	Model 1: Moral courage (total effect)		Model 2: Professional identity		Model 3: Moral courage (direct effect)	
	β	B (SE)	β	B (SE)	β	B (SE)
Predictors						
Moral sensitivity	**0.505**	1.442**^∗∗∗^** (0.747)	**0.328**	1.261**^∗∗∗^** (0.113)	**0.372**	1.062**^∗∗∗^** (0.070)
Professional identity		—		—	**0.407**	0.302**^∗∗∗^** (0.018)
Covariates						
Head nurse	**0.092**	4.688**^∗∗∗^** (1.414)	0.000	0.006 (2.139)	**0.092**	4.686**^∗∗∗^** (1.259)
Department						
Surgery	−0.004	−0.139 (1.092)	0.034	1.760 (1.651)	−0.018	−0.669 (0.973)
Gynecology	0.013	1.093 (2.274)	−0.022	−2.571 (3.439)	0.022	1.869 (2.025)
Paediatrics	0.049	3.011 (1.676)	0.008	6.196 (2.534)	0.046	2.824 (1.492)
Emergency	−0.020	−1.446 (2.001)	−0.029	−2.891 (3.027)	−0.008	−0.574 (1.783)
ICU	−0.002	−0.138 (1.700)	−0.021	−1.753 (2.571)	0.006	0.391 (1.513)
Operating room	**−0.063**	−7.275**^∗^** (3.071)	−0.008	−1.298 (4.644)	**−0.060**	−6.883**^∗^** (2.734)
Outpatient	−0.011	−0.632 (1.590)	−0.025	−1.864 (2.405)	−0.001	−0.069 (1.416)
Others	−0.005	−0.285 (1.499)	−0.019	−1.374 (2.267)	0.002	0.130 (1.334)
Average night shifts/month						
1–4	0.039	1.517 (1.184)	0.027	1.427 (1.790)	0.028	1.086 (1.054)
≥ 5	**−0.089**	−2.752**^∗∗^** (0.988)	−0.050	−2.083 (1.494)	**−0.069**	−2.123**^∗^** (0.880)
Model summary						
R	0.549		0.346		0.669	
*R* ^2^	0.302		0.120		0.447	
F	37.262		11.732		64.338	
Constant	21.632		64.584		2.147	

*Note:* B = unstandardized coefficient. Covariates included: position, department, and average night shifts per month. Position was dummy‐coded (0 = staff nurse, 1 = head nurse). Department was entered as a set of dummy variables (reference: Internal Medicine). Night shift frequency was dummy‐coded into two categories (reference: 0 shifts per month). Bold values indicate statistically significant coefficients (*p* < 0.05).

Abbreviation: SE, standard error.

^∗^ *p* < 0.05.

^∗∗^ *p* < 0.01.

^∗∗∗^ *p* < 0.001 (two‐tailed).

**TABLE 5:** Decomposition table of mediating effect.

Path effect	Effect size	Boot SE	LLCI	ULCI	Effect percentage (%)
Total effect	1.4424	0.075	1.296	1.589	100
Direct effect	1.0619	0.070	0.924	1.200	73.62
Indirect effect	0.3805	0.045	0.298	0.473	26.38

*Note:* The effect percentage was calculated as indirect effect ÷ total effect × 100%, using unstandardized coefficients (B values). Bootstrapping based on 5000 resamples. The proportion of the total effect explained by the indirect path should be interpreted with caution given the cross‐sectional correlational design.

Abbreviations: Boot SE, bootstrap standard error; LLCI, lower limit confident interval; ULCI, upper limit confident interval.

Moral sensitivity was positively associated with professional identity (*B* = 1.261, SE = 0.113, *β* = 0.328, *p* < 0.001) and, when both moral sensitivity and professional identity were entered simultaneously, both remained positively associated with moral courage (*B* = 1.062, SE = 0.070, *β* = 0.372, *p* < 0.001 for moral sensitivity; *B* = 0.302, SE = 0.018, *β* = 0.407, *p* < 0.001 for professional identity). The total effect of moral sensitivity on moral courage was also significant (*B* = 1.442, SE = 0.075, *β* = 0.505, *p* < 0.001).

As shown in Table 5, the indirect effect of moral sensitivity on moral courage through professional identity was 0.3805 (Boot SE = 0.045), with a 95% bias‐corrected bootstrap CI of 0.298 to 0.473. The indirect effect accounted for 26.38% of the total effect, indicating that professional identity partially mediated the relationship between moral sensitivity and moral courage. The direct and indirect effects accounted for 73.62% and 26.38% of the total effect, respectively. The final model is presented in Figure 1.

**FIGURE 1:**
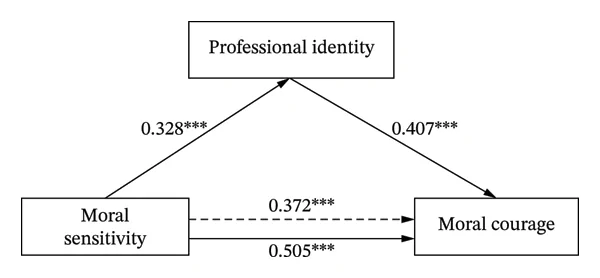
Final model. Note: path coefficients are standardized regression weights (β values). *p* < 0.001. The effect of moral sensitivity on professional identity was 0.328; the effect of professional identity on moral courage was 0.407; the direct effect of moral sensitivity on moral courage was 0.372; the total effect was 0.505.

## 4. Discussion

### 4.1. What Does This Study Add for Nurse Managers?

Findings from this cross‐sectional study offer three noteworthy observations relevant to nursing management. First, professional identity was significantly correlated with moral sensitivity and moral courage. The mediation analysis further confirmed a meaningful indirect association from moral sensitivity to moral courage mediated by professional identity. Second, the three key constructs correlated positively with one another, which implies they constitute interconnected dimensions of nurses’ ethical functioning; nevertheless, this inference awaits more empirical evidence. Third, operating‐room nurses and staff undertaking five or more monthly night shifts obtained relatively low moral courage scores. Such subgroups merit targeted attention in future research and clinical practice. Owing to the correlational nature of cross‐sectional data, our results can only generate testable hypotheses for follow‐up longitudinal or experimental studies. They do not confirm causal relations and cannot validate concrete management interventions.

### 4.2. Benchmarking Moral Courage: Implications for Management Expectations

The mean moral courage score in our sample (77.98/105, SD = 15.34) is comparable to previous studies of Chinese clinical nurses [5, 8, 38] but notably lower than scores reported among ICU nurses in Turkey during the COVID‐19 pandemic [39] and registered nurses in Finland [6]. For nurse managers, these cross‐national differences are consistent with the view that moral courage may be influenced by contextual factors—including organizational ethical climate, leadership practices, and professional recognition systems—that are, at least in part, amenable to managerial intervention. Rather than simply benchmarking against other populations, managers should assess baseline moral courage levels within their own units and identify unit‐specific barriers to courageous action.

In terms of demographic associations, head nurses reported significantly higher moral courage scores than staff nurses without leadership positions [40]. This finding is consistent with previous research showing that leadership roles are associated with more opportunities to exercise ethical decision‐making and advocacy [11]. Nurses working in paediatrics had the highest moral courage scores, whereas those in operating rooms had the lowest. One possible explanation is that pediatric nurses frequently encounter vulnerable patients and families, which may heighten their ethical awareness and willingness to act [41]. Conversely, operating room nurses often work within highly protocol‐driven environments that may limit opportunities for autonomous ethical decision‐making [42].

We also identified night shift frequency as a modifiable risk factor. Nurses working five or more night shifts per month reported significantly lower moral courage scores than those working 1–4 shifts (*p* < 0.001). This finding extends prior research linking shift work to ethical outcomes [43, 44] by identifying a specific dose–response pattern: moderate night shift exposure (1–4 shifts/month) may sustain ethical vigilance, while higher exposure (≥ 5 shifts/month) may erode moral courage through physical and emotional exhaustion. For nurse managers, this finding has clear operational implications: (a) Monitor cumulative night shift burden rather than simply counting total shifts; (b) Limit consecutive night shifts and ensure adequate recovery periods for nurses assigned to 5+ night shifts per month (c) Provide accessible ethics consultation (e.g., on‐call ethics phone line) during night hours when managerial oversight is reduced; and (d) Consider rotating high‐night‐shift nurses to day shifts periodically to restore ethical capacity.

### 4.3. Patterns of Association Between Moral Sensitivity, Professional Identity, and Moral Courage

Consistent with prior research [10, 45], moral sensitivity was positively correlated with moral courage in our sample. This finding is consistent with the theoretical premise that the ability to recognize ethical issues may be foundational to acting courageously upon them [46]. However, our correlation coefficient between moral sensitivity and moral courage (*r* = 0.517, *p* < 0.001) was slightly lower than that reported by Luo et al. (*r* = 0.613, *p* < 0.01) among nurse interns [8]. This difference may reflect the greater variability in clinical experience and ethical exposure among registered nurses compared to relatively homogeneous student populations. Additionally, professional identity was positively correlated with both moral sensitivity [47] and moral courage [24]. The stronger correlation between professional identity and moral courage suggests that identification with the nursing profession may be more directly relevant to courageous action than to ethical recognition, a finding consistent with the conceptualization of professional identity as a source of ethical motivation [24, 48].

### 4.4. How Professional Identity Bridges Sensitivity and Courage

Our mediation finding supports Rest’s four‐component model [17, 49] by empirically identifying professional identity as the moral motivation mechanism that translates ethical recognition into courageous action [50]. The partial mediation (26.38%), however, reveals a more nuanced picture: identity‐based motivation is important but does not fully account for the sensitivity‐courage link. The substantial direct effect suggests that other pathways operate alongside or instead of identity, including moral judgment (clarity about ethically sound actions reduces decisional conflict) [46], moral implementation (communication and assertiveness skills lower perceived barriers) [11], and contextual moderators such as ethical leadership and team support [5, 10]. These mechanisms may be particularly influential for moral courage, which—unlike routine ethical behavior—requires overcoming acute interpersonal or hierarchical risks [11]. This aligns with recent calls to view ethical action as emerging from dynamic interactions between individual dispositions and situational affordances, rather than a fixed linear progression [49]. For nurse managers, these refinements suggest that strengthening professional identity should be complemented by interventions targeting ethical reasoning, advocacy skills, and workplace climate [48].

## 5. Implications for Nursing Management

The finding that professional identity partially mediated the relationship between moral sensitivity and moral courage suggests three directions that nurse managers may consider when designing interventions to support ethical practice.

First, the mediating role of professional identity indicates that ethics training programmes could benefit from incorporating components that strengthen professional identity alongside cognitive case analysis. Reflective exercises that help nurses articulate the connection between their daily actions and professional values may be one way to address this.

Second, the subgroup differences observed in this study—particularly the lower moral courage scores among operating room nurses and nurses working five or more night shifts per month—suggest that these groups may benefit from targeted organizational support. Managers may wish to assess the specific barriers to courageous action in these settings and consider whether adjustments to work arrangements or additional ethical support during night hours could be helpful.

Third, the positive association between professional identity and moral courage implies that organizational recognition of ethical behavior could reinforce professional identity. Managers might explore whether simple recognition mechanisms, such as acknowledging courageous acts in team meetings or performance evaluations, can contribute to sustaining ethical practice.

These suggestions are based on correlational findings from a cross‐sectional study and should be interpreted as preliminary directions for further investigation. Their effectiveness in improving moral courage would need to be confirmed through future longitudinal or experimental research.

## 6. Limitations

A key limitation of this work originates from its single‐wave cross‐sectional design. Since all data were gathered at one time‐point, we cannot establish the temporal ordering among moral sensitivity, professional identity, and moral courage. Multiwave longitudinal investigations will be essential for untangling potential directional associations among these variables. Second, despite results from Harman’s single‐factor test, this study depends entirely on self‐report questionnaires filled out by the same respondents, so social‐desirability response biases cannot be eliminated. Our Harman analysis showed the leading single factor explained 42.45% of total variance. This result lies below the conventional 50% cutoff for severe common‐method bias, yet marginally surpasses the stricter 40% threshold used in some methodological papers. The gap is small (2.45 percentage points), indicating common‐method effects are unlikely to greatly bias our correlation estimates. Even so, future work may improve causal inference by adopting multisource ratings (for instance, peer or supervisor evaluations of moral courage) or longitudinal repeated‐measure designs. Third, participants were recruited exclusively from two tertiary‐level hospitals within one city in Northeast China, which constrains how far these findings can be generalized to other geographical locations or different healthcare contexts.

## 7. Conclusions

This study demonstrates that professional identity partially mediates the relationship between moral sensitivity and moral courage among clinical nurses, accounting for approximately one‐quarter of the total effect. This finding provides empirical support for Rest’s four‐component model of morality, in which professional identity is conceptualized as a mechanism of moral motivation that links ethical recognition to courageous action.

Future research should test these interventions using longitudinal or experimental designs. Studies are also needed to examine whether the mediating effect of professional identity holds across different healthcare systems, and whether factors such as ethical leadership, staffing ratios, or nurse autonomy moderate this pathway. Given the cross‐sectional nature of our data, these recommended strategies should be interpreted as preliminary directions for intervention rather than evidence‐based conclusions, and their effectiveness warrants confirmation through future interventional studies.

## Author Contributions

Yuanyuan Zhang contributed to the study conception and design and manuscript writing. Yujing Wang contributed to the study conception and design, data analysis, and manuscript writing.

## Funding

This research did not receive any specific grant from funding agencies in the public, commercial, or not‐for‐profit sectors.

## Disclosure

All authors reviewed and approved the final manuscript.

## Ethics Statement

Ethical approval was obtained from the Ethics Committee of the Hospital of Stomatology, China Medical University (Approval No.: K2026007). All procedures performed were in accordance with the ethical standards of the institutional and national research committee and with the 1964 Helsinki Declaration and its later amendments. Written informed consent was obtained from each participant through the online platform before proceeding to the questionnaire. Participation was voluntary, and confidentiality of the data was strictly maintained throughout the study.

## Consent

Please see the Ethics Statement.

## Conflicts of Interest

The authors declare no conflicts of interest.

## Data Availability

The data that support the findings of this study are available from the corresponding author upon reasonable request.
